# Cohort profile: The BiCoVac cohort - a nationwide Danish cohort to assess short and long-term symptoms following COVID-19 vaccination

**DOI:** 10.1007/s10654-025-01204-1

**Published:** 2025-02-07

**Authors:** Christina Bisgaard Jensen, Kristoffer Torp Hansen, Casper Mailund Nielsen, Stefan Nygaard Hansen, Henrik Nielsen, Charlotte Ulrikka Rask, Per Fink, Thomas Meinertz Dantoft, Torben Jørgensen, Bodil Hammer Bech, Sanne Møller Thysen, Dorte Rytter

**Affiliations:** 1 Department of Public Health, Aarhus University, Bartholins Allé 2, Aarhus, DK-8000 Denmark; 2 Department of Infectious Diseases, Aalborg University Hospital, Aalborg, DK-9100 Denmark; 3 Department of Clinical Medicine, Aalborg University, Aalborg, DK-9000 Denmark; 4 Department of Child and Adolescent Psychiatry, Aarhus University Hospital Psychiatry, Aarhus, DK-8200 Denmark; 5 Department of Clinical Medicine, Aarhus University, Aarhus, DK-8200 Denmark; 6 Research Clinic for Functional Disorders and Psychosomatics, Aarhus University Hospital, Aarhus, DK-8200 Denmark; 7 Center for Clinical Research and Prevention, Copenhagen University Hospital - Bispebjerg and Frederiksberg, Copenhagen, DK-2000 Denmark; 8 Department of Public Health, Faculty of Health and Medical Sciences, University of Copenhagen, Copenhagen, DK-2200 Denmark; 9 Department of Clinical Pharmacology, Copenhagen University Hospital– Bispebjerg and Frederiksberg, Copenhagen, DK-2400 Denmark

**Keywords:** Cohort profile, COVID-19, Vaccine, Adverse events, Non-specific symptoms, Epidemiology

## Abstract

BiCoVac is a population-based Danish cohort aiming to examine whether Coronavirus disease 2019 (COVID-19) vaccines are associated with non-specific symptoms beyond the specific protection of COVID-19. Data were collected by four questionnaire surveys between May 2021 and July 2022 and the questionnaire distribution was aligned with the Danish COVID-19 vaccination program. All surveys collected self-reported information on symptoms (e.g., headache, nausea, and fatigue). The baseline survey additionally gathered information on lifestyle and health. Survey data were combined with data from the Danish registers including information on COVID-19 vaccination and COVID-19 test results. A total of 911,613 (25% of all Danish citizens aged 16 to 65) were randomly sampled for the cohort and 252,401 initiated the baseline questionnaire. Of these, 59% (*n* = 149,070) participated in the 1st follow-up, 43% (*n* = 107,655) in the 2nd follow-up, and 25% (*n* = 63,737) in the 3rd follow-up. Women and individuals above 40 years of age were more likely to participate. Among vaccinated respondents, 25–38% reported moderate to severe immediate symptoms following COVID-19 vaccination, varying by vaccine doses. Females, younger individuals, and those with prior COVID-19 reported more immediate symptoms. Results of potential non-specific symptoms following COVID-19 vaccination did not reveal higher risk of involuntary movements among vaccinated individuals compared to unvaccinated individuals. Currently (December 2024), we are further investigating the effects of COVID-19 vaccines on other non-specific symptoms and exploring whether specific characteristics render some individuals more susceptible to report non-specific symptoms. In addition, long-term symptoms following COVID-19 are being investigated.

**Supplementary Information:**

The online version contains supplementary material available at 10.1007/s10654-025-01204-1.

## Introduction

The vaccines against the Coronavirus disease 2019 (COVID-19) caused by the severe acute respiratory syndrome coronavirus 2 (SARS-CoV-2) were introduced in December 2020 and have been used worldwide. By January 2024, approximately 70% of the world population and more than 81% of all Danes have received one or more doses of a COVID-19 vaccine [1]. In Denmark, the national implementation of the vaccine program was based on a risk assessment where individuals at elevated risk of COVID-19 were prioritized [2]. The first individuals who were invited for COVID-19 vaccination were the elderly and people at risk of developing severe illness with SARS-CoV-2 as well as their relatives. Employees in the healthcare sector, elderly care sector, and selected parts of the social sector were also prioritized. The vaccination program included two vaccine doses of either BNT162b2 (Pfizer-BioNTech), mRNA-1273 (Moderna), or ChAdOx1-S (AstraZeneca) (in March 2021 ChAdOx1-S was suspended as a reaction to reports of thromboembolic events) [3]. Later ChAdOx1-S and Ad26.COV2-S (Janssen) were offered as an optional scheme [4]. A third vaccine dose was introduced in the national vaccination program in August 2021 [5]. Starting in January 2022, severely immunocompromised individuals were offered a booster dose, and from September 2022 booster doses were offered annually for specific age groups and populations at high risk of developing severe illness with SARS-CoV-2 [6–8]. The target populations were continuously adjusted over time to ensure prioritized protection for those most vulnerable.

## Why was the cohort established?

The rapid introduction of the COVID-19 vaccines raised public concerns regarding potential adverse events [9]. Some transient adverse events are expected following vaccination, but symptoms unrelated to the vaccine could also occur in temporal relation to vaccination simply by chance. When the etiological mechanism underlying these symptoms is ‘unknown’, vaccination could be a tangible explanation, and the affected individuals may mistakenly conclude that the vaccine was the cause of their symptoms. This could particularly be the case for longer-lasting non-specific symptoms such as headaches, palpitations, nausea, muscle pain, and fatigue, which are common symptoms in the general population [10]. It is also possible that symptoms could be the result of psychological mechanisms e.g., a nocebo effect following vaccination. In addition, the symptoms could represent an adverse drug reaction. However, it can be difficult to disentangle the cause of the symptoms on an individual level. In Denmark, public concerns regarding potential adverse events also occurred when the human papillomavirus (HPV) vaccine was introduced to girls and young women, where reports of heterogeneous and common general non-specfic symptoms as suspected adverse events emerged and resulted in a marked decrease in vaccine uptake [11, 12]. Since the frequency of symptoms among those not vaccinated was not known (and data on this was scarce), it was however difficult to investigate if these symptoms appeared more often among vaccinated girls than expected from background rates.

The post-licensure surveillance of adverse events following COVID-19 vaccines set up by the Danish authorities includes spontaneous reporting of adverse events [13]. This information has been useful to obtain signals of serious adverse events (e.g., venous thromboembolic events), which were later tested using the Danish registers by comparing the incidence of the events in vaccinated compared with unvaccinated individuals using specific diagnosis codes [14]. However, spontaneous reports are only collected among those vaccinated and as the Danish registers do not entail much information on common general non-specific symptoms, information on the frequency of these symptoms before vaccination and for unvaccinated individuals is not captured. It is therefore challenging to determine whether the reported symptoms occur more frequently among vaccinated than unvaccinated individuals. To earn and maintain public trust, it is important to decipher which suspected adverse events are caused by the vaccine and which symptoms can be explained by other factors. It was, therefore, important to supplement the surveillance already set up by the authorities.

Consequently, a population-based cohort of Danish citizens “The BiCoVac Cohort” (Danish: Bivirkninger ved Corona Vacciner) was established to collect information on symptoms prior to and after COVID-19 vaccination. In addition, data was collected to explore whether specific characteristics (e.g., biological, physical, and psychological measures) render some individuals more susceptible to developing adverse events than others. A secondary aim was to compare the frequency of symptoms between individuals with prior and no prior COVID-19.

**Fig. 1:**
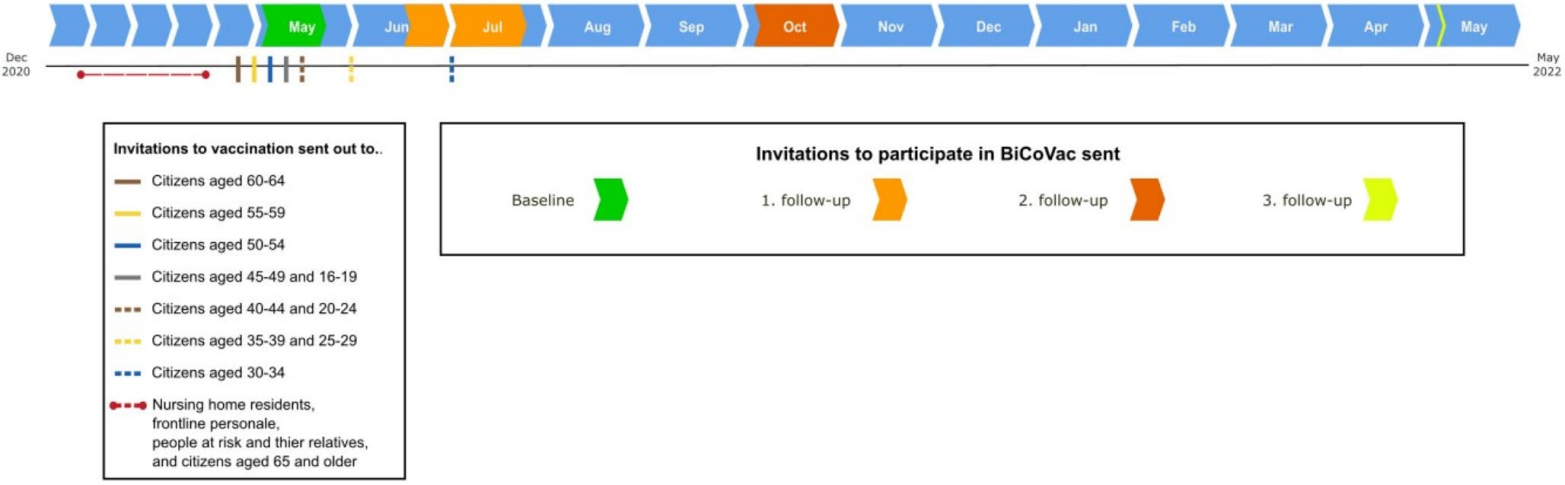
Distribution of questionnaires

**Fig. 2:**
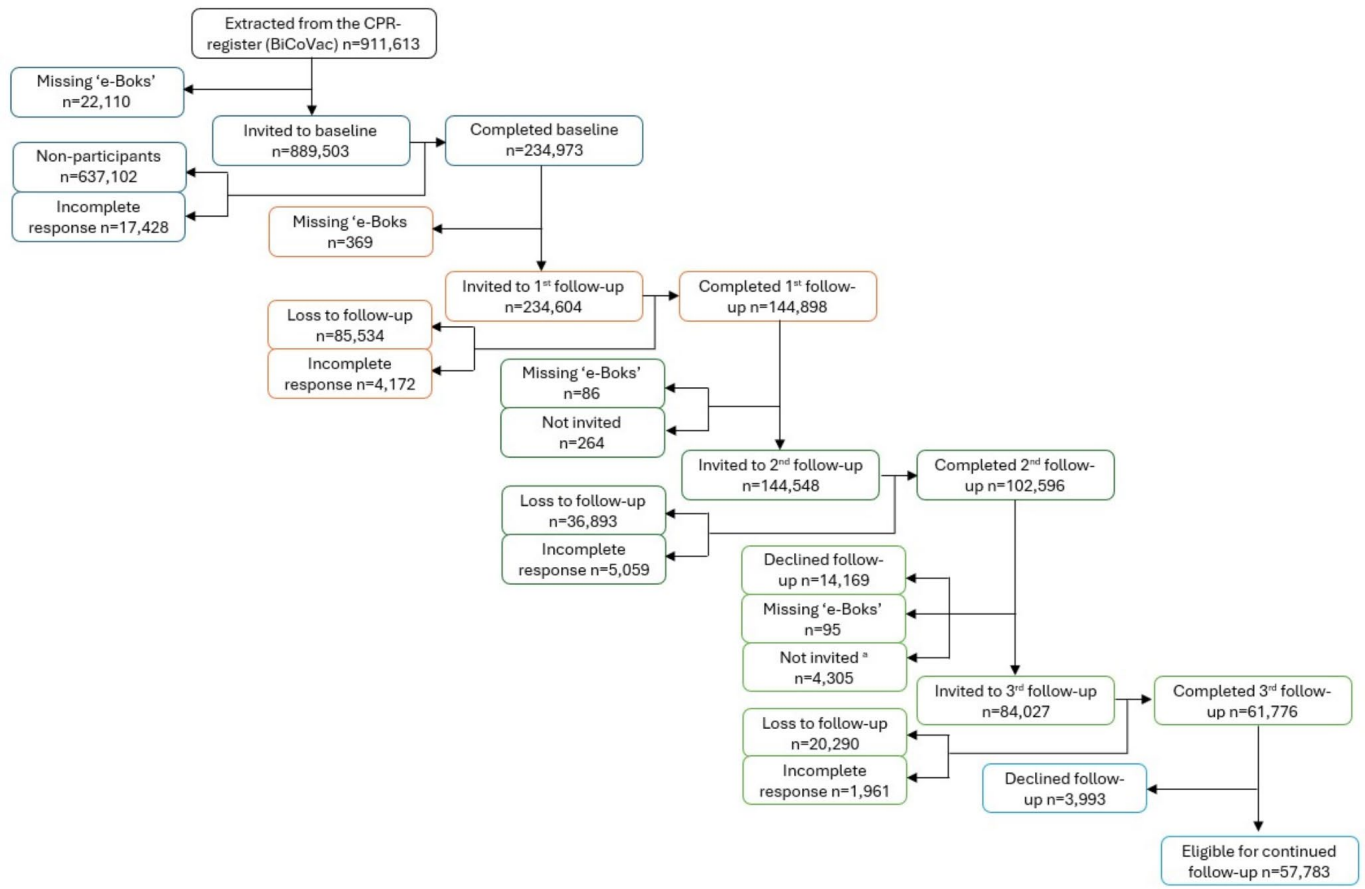
Flowchart. Legend: ^a^ Due to an error in the distribution of the 3^rd^ follow-up questionnaire, individuals aged 30-34 years were by mistake not invited

## Methods and materials

### How often has the cohort been followed up?

By January 2024, the BiCoVac cohort comprised four questionnaire surveys. The distribution of the questionnaires according to the national vaccination program is illustrated in Fig. 1. The baseline questionnaire was distributed in May 2021 before COVID-19 vaccines were given to the general population below 65 years of age to obtain background information before COVID-19 vaccination. The 1st and 2nd follow-up questionnaires were distributed according to the vaccination schedule: the 1st follow-up questionnaire was sent approximately two to four weeks after the age group was scheduled to complete the first dose of a COVID-19 vaccine (June-July 2021), whereas the 2nd follow-up questionnaire was sent approximately three months after the age group was scheduled for the 2nd dose (October 2021). A 3rd follow-up questionnaire was distributed approximately one year after the baseline questionnaire (May 2022).

**Table 1:** Categories of collected information in each questionnaire in the BiCoVac cohort

Categories	Baseline	1st follow-up	2nd follow-up	3rd follow-up
COVID-19 vaccination	X	X	X	X
Immediate local and systemic symptoms following COVID-19 vaccination ^a^	X	X	X	X
Vaccine target group (staff in the healthcare, elderly, or social sector; persons at elevated risk, relatives to persons at elevated risk; or others)	X			
COVID-19	X	X	X	X
Infections beyond COVID-19	X	X	X	X
25-item BDS checklist, involuntary muscle movements, sleep disturbance, and visual disturbances	X	X	X	X
Tinnitus, loss of sense of smell, and loss of sense of taste		X	X	X
Fatigue and exhaustion inspired by DePaul Symptom Questionnaire Short-Form-PEM ^b^		X	X	X
Shingles			X	X
Menstrual cycle			X	X
Overall health inspired by SF-12	X	X	X	
Comorbidity ^c^	X			
Mental health (SCL-13)	X			
COVID-19 vaccine hesitancy	X	X		
General health anxiety (Whiteley-6-R)	X			
Lifestyle (weight, height, physical activity level, alcohol intake, and smoking)	X			
Stress (Cohen’s 10-item PSS)	X			
Consent for continued follow-up			X	X

Abbreviation: BDS = Bodily distress syndrome. SCL-13 = 13-item Symptom Checklist. SF-12 = 12-item Short Form Survey. PSS = Perceived Stress Scale

Legend: ^a^ In the Danish version of the questionnaire, two symptoms (tiredness and fatigue) were asked very similarly and presumably reflected the same reaction

^b^ All five items were included but only the severity of the items were addressed. Further the time frame were altered to refer to the past four weeks

^c^ Co-morbidity included cancer, diabetes, hypertension, high cholesterol, heart attack, other heart disease, stroke, depression, anxiety disorder, asthma, hay fever, child eczema, psoriasis, osteoarthritis, celiac disease, osteoporosis, chronic obstructive pulmonary disease, fibromyalgia, irritable bowel syndrome, chronic fatigue syndrome, multiple chemical sensitivity, and whiplash

### What has been measured?

All surveys collected self-reported information on COVID-19 vaccination, COVID-19, and symptoms including immediate symptoms following vaccination, common general non-specific symptoms (such as headache, fatigue, and nausea), and COVID-19 severity. The baseline survey additionally collected self-reported information on general health, lifestyle, mental health, health anxiety, and COVID-19 vaccine hesitancy. Those vaccinated with COVID-19 vaccines were asked about the number of vaccine dose(s), type of vaccine(s), date(s) of vaccination(s), and 21 local and systemic symptoms in the immediate period following each COVID-19 vaccination (e.g., redness and/or pain at the injection site, fever, headache, pain in muscles and joints, bruises, and allergic reaction). These symptoms were reported as; ‘*None’*, ‘*Mild*’, ‘*Moderate*’, or *‘Severe’*. Individuals reporting prior COVID-19 were asked to report whether they were tested, the date of infection, and the degree of experienced symptoms (asymptomatic, mild, moderate, severe, or severe with hospitalization). We used validated questionnaires including the 25-item Bodily Distress Syndrome (BDS) checklist [15], the Symptom Checklist 13 (SCL-13) [16], Cohen’s Perceived Stress Scale (PSS) [17, 18], and the Whiteley-6-R scale [19]. Due to the emergence of new potential adverse events, the follow-up questionnaires were extended during the follow-up period to gather information on additional symptoms, such as tinnitus, shingles, and details regarding menstrual cycle characteristics. An overview of the collected information in each questionnaire can be found in Table 1. The questionnaires were available in Danish, English, and Arabic. For questions where official validated translations existed, the respective language versions were used. For the remaining questions, translation was conducted by a professional translator. The baseline and three follow-up questionnaires can be found in Supplementary File S1, S2, S3, and S4, respectively (Online Resource 1–4).

**Table 2:** List of most relevant linked registers

Register	Information
The Civil Person Registry	Demography such as sex, age, death, migration, ethnicity, country of origin, and municipality of residences as well as social context; households, families, children, and marital status
Surveillance data on COVID-19	COVID-19 vaccination type, doses, and dates. COVID-19 test results (Polymerase Chain Reaction (PCR) and antigen tests) and dates
The National Patient Register	In-patient and out-patient contacts to public and private somatic and psychiatric hospitals (hospital admissions, dates, admission type, diagnoses, contact type and reason, procedures, waiting time, and accident)
The Educational Attainment Register	Highest level of completed education
The Income Statistics Register	Income
Danish Register for Evaluation of Marginalization	Unemployment benefits, sickness absence, disability pension, early retirement, and regular retirement
The Registry of Causes of Death	Date and causes of death
Registry of Medicinal Product Statistics	Information on redeemed prescriptions
The National Health Insurance Service Registry	Contact and services in the primary health care
The Employment Classification Module	Occupation and employment

**Table 3:** Characteristics of the participants

	Random sample *N* (%)	Baseline *N* (%)	1st follow-up *N* (%)	2nd follow-up *N* (%)	3rd follow-up *N* (%)
**Total**	911,613 (100)	252,401 (28)	149,070 (16)	107,655 (12)	63,737 (7)
**Sex**					
Women	450,439 (49)	150,612 (60)	92,027 (62)	66,538 (62)	39,202 (62)
Men	461,174 (51)	101,789 (40)	57,043 (38)	41,117 (38)	24,535 (38)
**Age**					
16-29	243,501 (27)	38,180 (15)	15,119 (10)	8,231 (8)	3,942 (6)
30-50	380,882 (42)	93,494 (37)	51,316 (34)	34,261 (32)	16,957 (27)
51-65	287,230 (31)	120,727 (48)	82,635 (56)	65,163 (60)	42,838 (67)
**Education** ^a^					
Primary and upper secondary education	303,868 (33)	59,527 (24)	29,265 (20)	19,619 (18)	11,127 (17)
Short-cycle tertiary education	310,576 (34)	94,116 (37)	57,229 (38)	41,861 (39)	25,308 (40)
Medium cycle education and BA	168,457 (19)	58,890 (23)	37,716 (25)	27,902 (26)	16,839 (26)
Master or equivalent and PhD	117,120 (13)	38,015 (15)	23,980 (16)	17,679 (16)	10,147 (16)
*Missing information*	11,592 (1)	1,853 (1)	880 (1)	594 (1)	316 (1)
**Socioeconomic status of the family ** ^b^					
Self-employed/executive	86,627 (10)	27,091 (11)	16,162 (11)	11,876 (11)	7,373 (12)
Employed	639,786 (70)	188,006 (74)	111,897 (75)	80,614 (75)	47,347 (74)
On social benefits	113,442 (12)	25,589 (10)	15,418 (10)	11,646 (11)	7,113 (11)
Student	51,920 (6)	9,175 (4)	4,142 (3)	2,380 (2)	1,187 (2)
Other	19,481 (2)	2,517 (1)	1,444 (1)	1,139 (1)	717 (1)
*Missing information*	357 (<1)	23 (<1)	7 (<1)	NA	NA
**Origin** ^c^					
Danish	756,807 (83)	228,865 (91)	139,723 (94)	101,747 (95)	60,960 (96)
Western descendants	54,532 (6)	9,886 (4)	4,858 (3)	3,335 (3)	1,754 (3)
Western immigrants	2,624 (<1)	594 (<1)	313 (<1)	221 (<1)	126 (<1)
Non-western descendants	76,062 (8)	11,076 (4)	3,534 (2)	2,020 (2)	793 (1)
Non-western immigrants	21,436 (2)	1,955 (1)	630 (<1)	332 (<1)	104 (<1)
*Missing information*	152 (<1)	25 (<1)	12 (<1)	NA	NA
**No. of COVID-19 vaccines** **received**					
No vaccine		170,752 (68)	12,412 (8)	3,146 (3)	1,155 (2)
1st vaccine		47,618 (19)	71,144 (48)	740 (1)	103 (<1)
2nd vaccine		33,946 (13)	65,468 (44)	99,176 (92)	2,327 (4)
3rd vaccine		12 (<1)	20 (<1)	4,583 (4)	60,152 (94)
*Missing information*		73 (<1)	26 (<1)	10 (<1)	NA
**Vaccine type** ^d^					
BNT162b2		58,992 (72)	109,921 (80)	81,891 (78)	50,480 (81)
mRNA-1273		4,272 (5)	13,469 (10)	10,443 (10)	5,408 (8)
ChAdOx1-S		9,060 (11)	222 (<1)	71 (<1)	15 (<1)
Ad26.COV2-S		621 (1)	1,384 (1)	349 (<1)	15 (<1)
Mixed		8,631 (11)	11,636 (9)	11,745 (11)	6,664 (11)
*Missing information*		73 (<1)	26 (<1)	10 (<1)	NA
**COVID-19 ** ^e^					
No prior COVID-19		238,539 (95)	140,508 (94)	100,834 (94)	30,914 (49)
Prior COVID-19		13,862 (5)	8,562 (6)	6,821 (6)	32,823 (51)

Abbreviation: NA = Not applicable due to low numbers (<5). If participants had these characteristics, they were combined with one of the other categories

Legend: Characteristics were presented for the random sample as well as all of those who initiated each questionnaire. COVID-19 vaccine doses, vaccine type, and prior COVID-19 were handled as time-varying variables and therefore not provided for the random sample, while for the participants it was defined by the time, they initiated each questionnaire. All information was based on the Danish registers

^a^ Education was based on the highest level of educational attainment in 2021 and in case of missing information, we utilized data from the year before back to 2018

^b^ Socioeconomic status of the family was based on the highest income of the household in 2021 and in case of missing information, we utilized data from the year before back to 2019

^c^ Western origin included countries in the European Union as well as Andorra, Iceland, Liechtenstein, Monaco, Norway, San Marion, Switzerland, Great Britain, Vatican City State, Canada, United States of America, Australia, and New Zealand, while non-western origin includes the remaining countries

^d^ Vaccine type was only defined among those vaccinated against COVID-19

^e^ Prior COVID-19 was based on test results from Polymerase Chain Reaction (PCR) and antigen tests. A positive antigen test followed by a negative PCR test within 7 days was considered a false positive test result

The Danish Civil Personal Registration (CPR) identification (a unique 10-digit civil registration number assigned to all Danish citizens) allowed linkage to individual-level data from the national Danish registers. This included, among others, the National Patient Register and the Surveillance data on COVID-19 (COVID-19 test results and COVID-19 vaccines) monitored by Statens Serum Institut (a research institution under the Danish Ministry of Health) with the potential for further linkage to additional Danish registers e.g., The Laboratory Information Systems, The Danish Medical Birth Register, The Cancer Registry, and more. A list of the most relevant linked registers can be found in Table 2. All linked registers were made available by Statistics Denmark.

### Who is in the cohort?

The cohort consists of a sample of Danish citizens aged 16–65 years and living in Denmark in April 2021. In total, 911,613 were randomly sampled from the unique CPR System, stratified by year of birth (25% from each birth year of eligible individuals). Invitations were linked to the CPR identification and distributed through the national digital mailbox system ‘e-Boks’, used for communication between authorities and Danish citizens. A reminder was sent out 2–3 weeks after the initial invitation. More than 40% of invited participants above 50 years of age responded to the baseline questionnaire, whereas the participation rate among those younger than 30 years of age was less than 15%. A total of 252,401 (28%) citizens initiated the baseline questionnaire. Of these, 60% were women and 68% were unvaccinated at the time of initiating the baseline questionnaire. Participants who answered follow-up questionnaires were more often older, of Danish origin, from higher socioeconomic classes, and with longer education. At the end of the study period, 98% of the participants were vaccinated against COVID-19. Characteristics of the random sample and the participants who initiated each questionnaire can be seen in Table 3.

All participants who completed a questionnaire were invited to answer the following questionnaire (renewed consent was required to participate in the 3rd follow-up questionnaire). For those who participated in the baseline questionnaire, the participation rates for the follow-up questionnaires were: 1st follow-up: 59% (*n* = 149,070); 2nd follow-up: 43% (*n* = 107,655); 3rd follow-up: 25% (*n* = 63,737). Due to an error in the distribution of questionnaires in the 3rd follow-up, individuals aged 30–34 years were not invited to participate. Figure 2 contains a flow diagram of the participation in each questionnaire. Participation stratified by vaccine dose can be found in Supplementary Fig. S1 (Online Resource 5).

## Results

### What has been studied and found to date? Key findings and publications

Among the vaccinated participants in the BiCoVac cohort, 25–38% (depending on vaccine dose) reported moderate to severe immediate symptoms following COVID-19 vaccination [20]. Hence, most of the participants did not experience immediate symptoms. The most reported immediate symptoms included redness and/or pain at the injection site, tiredness, muscle pain, and headache. Females, younger individuals, and those with prior COVID-19 reported more immediate symptoms compared to males, older individuals, and those with no prior COVID-19. Participants, who received the ChAdOx1-S vaccine reported more immediate symptoms following the first vaccine dose compared with individuals who received other vaccine types. Across all vaccine doses, participants vaccinated with mRNA-1273 reported more immediate symptoms compared to those who received BNT162b2 [20]. Additionally, health anxiety and COVID-19 vaccine hesitancy influenced the reporting of immediate symptoms following vaccination, with the effect being particularly pronounced among individuals with COVID-19 vaccine hesitancy [21].

Current results of potential post-acute non-specific symptoms following COVID-19 vaccination did not reveal higher risk of involuntary movements among vaccinated individuals compared to unvaccinated individuals [22].

Among the menstruating women, 30% reported menstrual changes following COVID-19 vaccination [23]. Risk factors common for menstrual changes such as stress, age, and smoking were associated with reporting menstrual changes following COVID-19 vaccination. Also, women who were concerned about receiving a COVID-19 vaccine, with prior COVID-19, and/or had experienced other immediate symptoms following vaccination, were more likely to report menstrual changes following COVID-19 vaccination.

## Discussion

### Future plans

Several studies are planned using data from the BiCoVac cohort including investigating the associations between COVID-19 vaccination and longer-lasting non-specific symptoms. It will also be investigated whether specific pre-vaccination characteristics render some individuals more susceptible to develop both immediate and longer-lasting non-specific symptoms following vaccination. In addition to the studies on COVID-19 vaccines, the BiCoVac cohort will be used to assess the effect of COVID-19 on reported symptoms, where we can include data from both registered PCR test, antigen test, as well as self-reported COVID-19 to account for home-test. Further selection weights and alternative approaches/designs that can benefit the analyses will be considered and inplemented where applicable in all future studies.

Though the cohort is unique in its ability to assess adverse events following COVID-19 vaccination, this large and diverse cohort holds potential beyond COVID-19. The comprehensive background data and follow-up though national registries, provides several posibilites including exploreing prevalence and long-term consequences of dimensions such as mental health and general health symptoms.

Furthermore, a total of 57,783 individuals have given their consent for continued follow-up, which enables the possibility of a 4th follow-up.

### Strengths and limitations

The data from the BiCoVac cohort is a unique resource. Particularly due to the timing of the data collection, which coincided with the national vaccination programme, as well as the collection of symptoms both pre- and post-COVID-19 vaccination. Also, common general symptoms are not captured in the Danish registers, and therefore often not included in nationwide studies. This cohort provides the possibility for a more complete understanding of post-vaccination effects. In addition, the baseline questionnaire contained comprehensive information on many background and pre-vaccination factors, and more information is available by linkage to the national Danish registers. Thus, it is possible to control for a variety of relevant potential cofounders. The national registers further allow us to follow the participants. Furthermore, the large sample size and the random sampling of the BiCoVac cohort are important strengths.

The BiCoVac cohort also has some limitations. The response rate at baseline was low and although the aim was to distribute the questionnaires according to the Danish vaccination program this was not possible for each individual because the vaccine schedules were continuously adapted. Furthermore, 32% of the participants were already vaccinated at the time, they responded to the baseline questionnaire, with specific groups such as health professionals and people at risk of developing severe illness with SARS-CoV-2 being among those prioritized for vaccination. This increases the risk of selection bias and may affect the generalisability of results to a general Danish population. However, despite the low response rate at baseline, the sample size is large, and the national registers provide the possibility of exploring additional selection in the cohort. This enables the estimation and application of inverse probability of selection weights. Further, some questions were only included in the later questionnaires and some questions were only given to participants if they were vaccinated (e.g., menstrual cycle characteristics in the 2nd follow-up). Also, certain questions, such as those related to lifestyle, mental health, and vaccine hesitancy, were included exclusively in the first or the first and second questionnaires. As a result, these variables cannot be used to explore changes over time. The error in the distribution of the 3rd follow-up among individuals 30–34 years of age is also a limitation.

## Electronic supplementary material

Below is the link to the electronic supplementary material.

Supplementary Material 1

Supplementary Material 2

Supplementary Material 3

Supplementary Material 4

Supplementary Material 5

## Data Availability

The dataset is not publicly available due to national data security legislation on sensitive personal data. License to the data will be under the conditions stipulated by the Danish Data Protection Agency and Aarhus University.
