# Supplementary material for: Cohort profile: The BiCoVac cohort - a nationwide Danish cohort to assess short and long-term symptoms following COVID-19 vaccination
Source: Eur J Epidemiol. 2025 Feb 7;40(2):225–33. doi: 10.1007/s10654-025-01204-1 (PMC12018486; doi:10.1007/s10654-025-01204-1)
Supplement: Supplementary file 1 — Supplementary Material 1 [file 10654_2025_1204_MOESM1_ESM.docx]

**Supplementary File S1 – Baseline questionnaire**

**Title:** Cohort Profile: The BiCoVac Cohort - a nationwide Danish cohort to assess short and long-term symptoms following COVID-19 vaccination

**Journal name:** European Journal of Epidemiology

**Authors:** Christina Bisgaard Jensen, Kristoffer Torp Hansen, Casper Mailund Nielsen, Stefan Nygaard Hansen, Henrik Nielsen, Charlotte Ulrika Rask, Per Fink, Thomas Meinertz Dantoft, Torben Jørgensen, Bodil Hammer Bech, Sanne Møller Thysen, Dorte Rytter

**Affiliation of the corresponding author:** Department of Public Health, Aarhus University, DK-8000 Aarhus, Denmark

**E-mail of the corresponding author:** cbj@ph.au.dk

**Dear participant**

Thank you for taking the time to complete this questionnaire. All the questions you answer provide important knowledge, which can contribute to understanding whether corona vaccines are associated with side effects.

Your answers are important – regardless of whether you are healthy or ill –and whether you are planning on being vaccinated or not.

We ask you to answer all the questions as well as you can.

Your answers are saved continuously and are thus included in the survey, also if the questionnaire is only partially completed. However, you can change your answers prior to clicking on "Finish".

**Questions about vaccination against COVID-19**

*The following questions concern vaccination against COVID-19.*

**Were you or are you worried about being vaccinated?**

- No, not significantly concerned
- I am sceptical about it, but plan to be/have been vaccinated anyway
- I am concerned and do not plan to be vaccinated
- Unsure/do not wish to answer

**Have you been vaccinated against COVID-19?**

*Select Yes if you have received either the first vaccine or both the first and second vaccine.*

- Yes, I've been vaccinated once
- Yes, I've been vaccinated twice
- No

**Which vaccine did you receive?**

- Pfizer-BioNTech
- Moderna
- AstraZeneca
- Johnson & Johnson
- Other: _____________________________________
- Don’t know

**Which vaccine did you receive the first time?**

- Pfizer-BioNTech
- Moderna
- AstraZeneca
- Johnson & Johnson
- Other: _____________________________________
- Don’t know

**Which vaccine did you receive the second time?**

- Pfizer-BioNTech
- Moderna
- AstraZeneca
- Johnson & Johnson
- Other: _____________________________________
- Don’t know

**When did you receive the first vaccine?**

*If you do not remember the exact day you received the 1st vaccine, please just state the month you were vaccinated.*

| Day | Month | Year |  |
| --- | --- | --- | --- |
| - 1 | - January | - 2021 | - Do not remember |
| - 2 | - February | - 2020 |  |
| - 3 | - March | - 2019 |  |
| - 4 | - April |  |  |
| - 5 | - May |  |  |
| - 6 | - June |  |  |
| - 7 | - July |  |  |
| - 8 | - August |  |  |
| - 9 | - September |  |  |
| - 10 | - October |  |  |
| - 11 | - November |  |  |
| - 12 | - December |  |  |
| - 13 |  |  |  |
| - 14 |  |  |  |
| - 15 |  |  |  |
| - 16 |  |  |  |
| - 17 |  |  |  |
| - 18 |  |  |  |
| - 19 |  |  |  |
| - 20 |  |  |  |
| - 21 |  |  |  |
| - 22 |  |  |  |
| - 23 |  |  |  |
| - 24 |  |  |  |
| - 25 |  |  |  |
| - 26 |  |  |  |
| - 27 |  |  |  |
| - 28 |  |  |  |
| - 29 |  |  |  |
| - 30 |  |  |  |
| - 31 |  |  |  |

**Did you experience any of the following symptoms during the week after the first vaccination?***If less than one week has passed since you were vaccinated, please just indicate the symptoms you have experienced so far.*

|  | No | Yes, mild symptoms | Yes, moderate symptoms | Yes,  severe symptoms |
| --- | --- | --- | --- | --- |
| Redness and/or pain at the injection site |  |  |  |  |
| Skin rash |  |  |  |  |
| Nausea |  |  |  |  |
| Vomiting |  |  |  |  |
| Fever |  |  |  |  |
| Shivering/chills |  |  |  |  |
| Tiredness |  |  |  |  |
| General malaise |  |  |  |  |
| Joint pain |  |  |  |  |
| Muscle pain |  |  |  |  |
| Headache |  |  |  |  |
| Diarrhoea |  |  |  |  |
| Dizziness |  |  |  |  |
| Urge to sleep/fatigue |  |  |  |  |
| Swollen lymph nodes |  |  |  |  |
| Facial swelling |  |  |  |  |
| Facial paralysis |  |  |  |  |
| Pain in the arms and legs |  |  |  |  |
| Allergic reaction |  |  |  |  |
| Shortness of breath |  |  |  |  |
| Bruising/bleeding under the skin |  |  |  |  |

**When did you receive the second vaccine?**

*If you do not remember the exact day you received the 2nd vaccine, please just state the month you were vaccinated.*

| Day | Month | Year |  |
| --- | --- | --- | --- |
| - 1 | - January | - 2021 | - Do not remember |
| - 2 | - February | - 2020 |  |
| - 3 | - March | - 2019 |  |
| - 4 | - April |  |  |
| - 5 | - May |  |  |
| - 6 | - June |  |  |
| - 7 | - July |  |  |
| - 8 | - August |  |  |
| - 9 | - September |  |  |
| - 10 | - October |  |  |
| - 11 | - November |  |  |
| - 12 | - December |  |  |
| - 13 |  |  |  |
| - 14 |  |  |  |
| - 15 |  |  |  |
| - 16 |  |  |  |
| - 17 |  |  |  |
| - 18 |  |  |  |
| - 19 |  |  |  |
| - 20 |  |  |  |
| - 21 |  |  |  |
| - 22 |  |  |  |
| - 23 |  |  |  |
| - 24 |  |  |  |
| - 25 |  |  |  |
| - 26 |  |  |  |
| - 27 |  |  |  |
| - 28 |  |  |  |
| - 29 |  |  |  |
| - 30 |  |  |  |
| - 31 |  |  |  |

**Did you experience any of the following symptoms during the week after the second vaccination?**

*If less than one week has passed since you were vaccinated, please just indicate the symptoms you have experienced so far.*

|  | No | Yes, mild symptoms | Yes, moderate symptoms | Yes,  severe symptoms |
| --- | --- | --- | --- | --- |
| Redness and/or pain at the injection site |  |  |  |  |
| Skin rash |  |  |  |  |
| Nausea |  |  |  |  |
| Vomiting |  |  |  |  |
| Fever |  |  |  |  |
| Shivering/chills |  |  |  |  |
| Tiredness |  |  |  |  |
| General malaise |  |  |  |  |
| Joint pain |  |  |  |  |
| Muscle pain |  |  |  |  |
| Headache |  |  |  |  |
| Diarrhoea |  |  |  |  |
| Dizziness |  |  |  |  |
| Urge to sleep/fatigue |  |  |  |  |
| Swollen lymph nodes |  |  |  |  |
| Facial swelling |  |  |  |  |
| Facial paralysis |  |  |  |  |
| Pain in the arms and legs |  |  |  |  |
| Allergic reaction |  |  |  |  |
| Shortness of breath |  |  |  |  |
| Bruising/bleeding under the skin |  |  |  |  |

**Which vaccination target group(s) do you belong to?**

*You may tick more than one box*

- Staff in the following sectors - healthcare, elderly and selected parts of the social sector - with a particular risk of infection, or who perform a critical function
- Selected persons with conditions and diseases that give rise to a significantly increased risk of serious illness with COVID-19
- Relatives of persons with a significantly increased risk of serious illness with COVID-19 or relatives who are indispensable carers or care personnel
- Other

**Questions about infections**

*The following questions concern infections*

**Giving your best guess, do you think/know whether you have ever been infected with COVID-19?**

- No, I do not think I have been infected with COVID-19
- Yes, I think/know that I have been infected with COVID-19

**When did you test positive?**

*Please submit date.* *If you do not remember the exact day you tested positive for COVID-19, please just state the month and year you tested positive.*

| Day | Month | Year |  |
| --- | --- | --- | --- |
| - 1 | - January | - 2021 | - I was not tested |
| - 2 | - February | - 2020 | - Don’t remember |
| - 3 | - March | - 2019 |  |
| - 4 | - April |  |  |
| - 5 | - May |  |  |
| - 6 | - June |  |  |
| - 7 | - July |  |  |
| - 8 | - August |  |  |
| - 9 | - September |  |  |
| - 10 | - October |  |  |
| - 11 | - November |  |  |
| - 12 | - December |  |  |
| - 13 |  |  |  |
| - 14 |  |  |  |
| - 15 |  |  |  |
| - 16 |  |  |  |
| - 17 |  |  |  |
| - 18 |  |  |  |
| - 19 |  |  |  |
| - 20 |  |  |  |
| - 21 |  |  |  |
| - 22 |  |  |  |
| - 23 |  |  |  |
| - 24 |  |  |  |
| - 25 |  |  |  |
| - 26 |  |  |  |
| - 27 |  |  |  |
| - 28 |  |  |  |
| - 29 |  |  |  |
| - 30 |  |  |  |
| - 31 |  |  |  |

**Did you experience symptoms of the COVID-19 infection?**

- No
- Yes, but only mild symptoms
- Yes, moderate symptoms
- Yes, severe symptoms but without hospitalisation
- Yes, severe symptoms that resulted in hospitalisation

**Within the past four weeks, have you had an infection that you do not think was COVID-19?**

*For example, a cold, tonsillitis or bladder infections.*

- No
- Yes

**Questions about health, well-being, leisure time and lifestyle**

**In general, would you say your health is:**

| Excellent | Very good | Good | Fair | Poor |
| --- | --- | --- | --- | --- |
|  |  |  |  |  |

**Does your health now limit you in moderate activities such as moving a table, vacuuming or riding a bike?**

| No, not limited at all | Yes, slightly limited | Yes, very limited |
| --- | --- | --- |
|  |  |  |

**Does your health now limit you in climbing several flights of stairs?**

| No, not limited  at all | Yes, slightly limited | Yes, very limited |
| --- | --- | --- |
|  |  |  |

**During the past four weeks, have you had any of the following problems with your work or other regular daily activities as a result of your physical health?**

|  | At no point in time | Occasionally | Some of the time | Most  of the time | All the time |
| --- | --- | --- | --- | --- | --- |
| I accomplished less than I would like |  |  |  |  |  |
| I have been limited in the kind of work or other activities I have been able to perform |  |  |  |  |  |

**During the past four weeks, have you had any of the following problems with your work or other regular daily activities as a result of any emotional problems?**

|  | At no point in time | Occasionally | Some of the time | Most  of the time | All the time |
| --- | --- | --- | --- | --- | --- |
| I accomplished less than I would like |  |  |  |  |  |
| I didn´t do work or other activities as carefully as usual |  |  |  |  |  |

**During the past 4 weeks, how much did physical pain interfere with your normal work (both work outside the home and housework)?**

| Not at all | A little bit | Moderately | Quite a bit | Extremely |
| --- | --- | --- | --- | --- |
|  |  |  |  |  |

**Has a medical doctor ever told you that you had/have one or more of the following diseases or conditions?**

|  | No | Yes | Don’t know |
| --- | --- | --- | --- |
| Cancer |  |  |  |
| Diabetes |  |  |  |
| High blood pressure (hypertension) |  |  |  |
| High cholesterol |  |  |  |
| Heart attack |  |  |  |
| Other heart disease |  |  |  |
| Stroke |  |  |  |
| Depression |  |  |  |
| Anxiety disorder |  |  |  |
| Asthma |  |  |  |
| Hay fever |  |  |  |
| Child eczema (Asthma-eczema) |  |  |  |
| Psoriasis |  |  |  |
| Osteoarthritis |  |  |  |
| Gluten intolerance (celiac disease) |  |  |  |
| Osteoporosis |  |  |  |
| COPD/Smoker's lungs |  |  |  |
| Fibromyalgia |  |  |  |
| Irritable Bowel Disease |  |  |  |
| Chronic fatigue syndrome |  |  |  |
| Multiple chemical sensitivity (MCS) |  |  |  |
| Whiplash |  |  |  |

**How tall are you in cm (without shoes)?**

*E.g. 171 cm*

___________________________________________ cm

- Do not wish to answer

**What is your weight in kilograms?**

*Without clothes*

___________________________________________ kilograms

- Do not wish to answer

**Regarding physical activity in your leisure time, which of the following groups does, in your opinion, best describe your situation?**

- As a rule, you sit and read, watch TV, go to the cinema and spend your leisure time undertaking sedentary pursuits
- You go for walks, ride a bike a little or are physically active for at least four hours a week (light recreational building, domestic work, table tennis and bowling)
- You actively participate in sporting activities at least three times a week. If you do not practice a sport, but often perform heavy gardening or heavy leisure work, you will also be part of this group.
- You practice competitive sport or long-distance running several times a week

**How many units of alcohol (beer, wine or spirits) did you drink in total during the last week?**

*1 unit corresponds to: 1 beer (33 cl.), 1 glass of wine (12 cl.), 1 glass of dessert wine (8 cl.) or 1 glass of spirits (4 cl.)*

___________________________________________ units

**Do you smoke?**

- No, I have never smoked
- No, but I have previously smoked
- Yes, occasionally (less than 1 cigarette, cheroot, cigar or full pipe daily)
- Yes, daily

**Questions about symptoms you have been bothered by over the past four weeks**

**During the past four weeks, how much have you been bothered by?**

Symptoms from the heart and lungs

|  | Not at all | A bit | Somewhat | Quite  a bit | A lot |
| --- | --- | --- | --- | --- | --- |
| Palpitations or heart pounding? |  |  |  |  |  |
| Precordial discomfort? |  |  |  |  |  |
| Breathlessness without exertion? |  |  |  |  |  |
| Hyperventilation? |  |  |  |  |  |
| Hot or cold sweats? |  |  |  |  |  |
| Dry mouth? |  |  |  |  |  |

Symptoms from the stomach and the intestines

|  | Not at all | A bit | Somewhat | Quite  a bit | A lot |
| --- | --- | --- | --- | --- | --- |
| Frequent, loose bowel movements? |  |  |  |  |  |
| Abdominal pain? |  |  |  |  |  |
| Feeling bloated/full of gas/distended? |  |  |  |  |  |
| Diarrhoea? |  |  |  |  |  |
| Regurgitations? |  |  |  |  |  |
| Nausea? |  |  |  |  |  |
| Burning sensation of the chest or upper part of the stomach/epigastrium? |  |  |  |  |  |

Symptoms from muscles and joints

|  | Not at all | A bit | Somewhat | Quite  a bit | A lot |
| --- | --- | --- | --- | --- | --- |
| Pain in arms or legs? |  |  |  |  |  |
| Muscular aches or pain? |  |  |  |  |  |
| Pain in the joints? |  |  |  |  |  |
| Feeling of paralysis in the arms or legs? |  |  |  |  |  |
| Back ache? |  |  |  |  |  |
| Pain moving from one place to another? |  |  |  |  |  |
| Unpleasant numbness or tingling sensations? |  |  |  |  |  |

General symptoms

|  | Not at all | A bit | Somewhat | Quite  a bit | A lot |
| --- | --- | --- | --- | --- | --- |
| Concentration difficulties? |  |  |  |  |  |
| Excessive fatigue? |  |  |  |  |  |
| Headache? |  |  |  |  |  |
| Impairment of memory? |  |  |  |  |  |
| Dizziness? |  |  |  |  |  |

Other symptoms

|  | Not at all | A bit | Somewhat | Quite  a bit | A lot |
| --- | --- | --- | --- | --- | --- |
| Involuntary muscle movements/convulsions? |  |  |  |  |  |
| Sleep disturbances? |  |  |  |  |  |
| Visual disturbances? |  |  |  |  |  |

**In the most recent questions, you have ticked one or more physical symptoms in the last four weeks.**

**How much does your symptoms affect your life?**

| No affect at all | |  |  |  |  |  |  |  |  | Severely affects my life | |
| --- | --- | --- | --- | --- | --- | --- | --- | --- | --- | --- | --- |
| - 1 | - 2 | | - 3 | - 4 | - 5 | - 6 | - 7 | - 8 | - 9 | | - 10 |

**How long do you think your symptoms will continue?**

| A very short time | |  |  |  |  |  |  |  |  | Forever | |
| --- | --- | --- | --- | --- | --- | --- | --- | --- | --- | --- | --- |
| - 1 | - 2 | | - 3 | - 4 | - 5 | - 6 | - 7 | - 8 | - 9 | | - 10 |

**How much control do you feel you have over your symptoms?**

| Absolutely no control | |  |  |  |  |  |  |  |  | Extreme amount of control | |
| --- | --- | --- | --- | --- | --- | --- | --- | --- | --- | --- | --- |
| - 1 | - 2 | | - 3 | - 4 | - 5 | - 6 | - 7 | - 8 | - 9 | | - 10 |

**How much do you think that treatment can help your symptoms?**

| Not at all | |  |  |  |  |  |  |  |  | Extremely helpful | |
| --- | --- | --- | --- | --- | --- | --- | --- | --- | --- | --- | --- |
| - 1 | - 2 | | - 3 | - 4 | - 5 | - 6 | - 7 | - 8 | - 9 | | - 10 |

**How concerned are you about your symptoms?**

| Not at all concerned | |  |  |  |  |  |  |  |  | Extremely concerned | |
| --- | --- | --- | --- | --- | --- | --- | --- | --- | --- | --- | --- |
| - 1 | - 2 | | - 3 | - 4 | - 5 | - 6 | - 7 | - 8 | - 9 | | - 10 |

**How well do you feel you understand your symptoms?**

| Do not understand them at all | |  |  |  |  |  |  |  |  | Understand them very clearly | |
| --- | --- | --- | --- | --- | --- | --- | --- | --- | --- | --- | --- |
| - 1 | - 2 | | - 3 | - 4 | - 5 | - 6 | - 7 | - 8 | - 9 | | - 10 |

**How much does your symptoms affect you emotionally? (I.e. do they make you feel angry, afraid, restless or depressed?)**

| Not at all affected emotionally | |  |  |  |  |  |  |  |  | Extremely affected emotionally | |
| --- | --- | --- | --- | --- | --- | --- | --- | --- | --- | --- | --- |
| - 1 | - 2 | | - 3 | - 4 | - 5 | - 6 | - 7 | - 8 | - 9 | | - 10 |

**Questions about worrying about illness**

**During the past four weeks, how much have you been bothered by?**

|  | Not at all | A little | Some | To a great extent | Very much |
| --- | --- | --- | --- | --- | --- |
| Worrying that there is something seriously wrong with your body? |  |  |  |  |  |
| Worrying about whether you suffer from a disease that you have heard or read about? |  |  |  |  |  |
| Worrying about whether you suffer from a serious illness? |  |  |  |  |  |
| Thinking that the doctor might be wrong if he/she says that there is nothing to worry about? |  |  |  |  |  |
| Worrying about your health? |  |  |  |  |  |
| Recurring thoughts that there is something wrong with you that you find difficult to stop thinking about? |  |  |  |  |  |

**Questions about discomforts you have experienced within the past four weeks**

*Below are a number of issues and discomforts that a person may sometimes have. Please read each one carefully. Once you have done this, please tick the box that best describes the extent to which the problem in question has caused you discomfort during the past four weeks.*

**To what extent have you been bothered by:**

|  | Not at all | A bit | Some | To a great extent | Very much |
| --- | --- | --- | --- | --- | --- |
| Nervousness or inner unrest? |  |  |  |  |  |
| Thoughts about ending your life? |  |  |  |  |  |
| A feeling of being trapped? |  |  |  |  |  |
| Suddenly becoming afraid for no reason? |  |  |  |  |  |
| Self-criticism? |  |  |  |  |  |
| Feeling lonely? |  |  |  |  |  |
| Feeling blue? |  |  |  |  |  |
| Worrying too much? |  |  |  |  |  |
| Feeling anxious? |  |  |  |  |  |
| Feeling there is no hope for the future? |  |  |  |  |  |
| Feeling that everything is an effort? |  |  |  |  |  |
| Outbursts of terror or panic? |  |  |  |  |  |
| A feeling of worthlessness? |  |  |  |  |  |

**Questions about everyday stress**

*The questions concern your feelings and thoughts within the past four weeks. For each question, please tick the box to indicate how often you felt or thought in the way stated.*

**How often during the past four weeks:**

|  | Never | Almost never | Occasionally | Often | Very often |
| --- | --- | --- | --- | --- | --- |
| Have you been upset about something that happened unexpectedly? |  |  |  |  |  |
| Have you felt that you could not control the important things in your life? |  |  |  |  |  |
| Have you felt nervous and "stressed"? |  |  |  |  |  |
| Have you felt confident about your ability to cope with your personal problems? |  |  |  |  |  |
| Have you felt that things were going your way? |  |  |  |  |  |
| Have you felt that you could not cope with all the things you had to do? |  |  |  |  |  |
| Have you been able to control irritations in your life? |  |  |  |  |  |
| Have you felt that you were on top of things? |  |  |  |  |  |
| Have you been angered because of things that were outside of your control? |  |  |  |  |  |
| Have you felt difficulties were piling up so high that you could not overcome them? |  |  |  |  |  |

**That was the final question.**

Thank you for taking the time to answer the questionnaire.

When you close the questionnaire, it will no longer be possible to change your answers.

We will send you a short questionnaire in a few weeks.
