# Supplementary material for: Cohort profile: The BiCoVac cohort - a nationwide Danish cohort to assess short and long-term symptoms following COVID-19 vaccination
Source: Eur J Epidemiol. 2025 Feb 7;40(2):225–33. doi: 10.1007/s10654-025-01204-1 (PMC12018486; doi:10.1007/s10654-025-01204-1)
Supplement: Supplementary file 2 — Supplementary Material 2 [file 10654_2025_1204_MOESM2_ESM.docx]

**Supplementary File S2 – First follow-up questionnaire**

**Title:** Cohort Profile: The BiCoVac Cohort - a nationwide Danish cohort to assess short and long-term symptoms following COVID-19 vaccination

**Journal name:** European Journal of Epidemiology

**Authors:** Christina Bisgaard Jensen, Kristoffer Torp Hansen, Casper Mailund Nielsen, Stefan Nygaard Hansen, Henrik Nielsen, Charlotte Ulrika Rask, Per Fink, Thomas Meinertz Dantoft, Torben Jørgensen, Bodil Hammer Bech, Sanne Møller Thysen, Dorte Rytter

**Affiliation of the corresponding author:** Department of Public Health, Aarhus University, DK-8000 Aarhus, Denmark

**E-mail of the corresponding author:** cbj@ph.au.dk

**Dear participant**

Thank you for taking the time to complete this questionnaire, which is the second of a total of 3 questionnaires. All your answers provide important knowledge that can help our understanding of, whether corona vaccines are associated with side effects.

Your answers are important - whether you are healthy or sick - and whether you have been / are planning to be vaccinated or not. We ask you to complete all questions as well as you can.

Your answers are saved continuously and are thus included in the survey, also if the questionnaire is only partially completed. However, you can change your answers until you have pressed "Finish".

**Questions about vaccination against COVID-19**

*The following questions concern vaccination against COVID-19.*

**Were you or are you worried about being vaccinated?**

- No, not significantly concerned
- I am sceptical about it, but plan to be/have been vaccinated anyway
- I am concerned and do not plan to be vaccinated
- Unsure/do not wish to answer

**Have you been vaccinated against COVID-19?**

*Select Yes if you have received either the first vaccine or both the first and second vaccine.*

- Yes, I've been vaccinated once
- Yes, I've been vaccinated twice
- No

**Which vaccine did you receive?**

- Pfizer-BioNTech
- Moderna
- AstraZeneca
- Johnson & Johnson
- CureVac
- Novavax
- Other: _____________________________________
- Don’t know

**Which vaccine did you receive as your first dose?**

- Pfizer-BioNTech
- Moderna
- AstraZeneca
- Johnson & Johnson
- CureVac
- Novavax
- Other: _____________________________________
- Don’t know

**Which vaccine did you receive as your second dose?**

- Pfizer-BioNTech
- Moderna
- AstraZeneca
- Johnson & Johnson
- CureVac
- Novavax
- Other: _____________________________________
- Don’t know

**When did you receive the first vaccine?**

*If you do not remember the exact day you received the 1st vaccine, please just state the month you were vaccinated.*

| Day | Month | Year |  |
| --- | --- | --- | --- |
| - 1 | - January | - 2021 | - Do not remember |
| - 2 | - February | - 2020 |  |
| - 3 | - March | - 2019 |  |
| - 4 | - April |  |  |
| - 5 | - May |  |  |
| - 6 | - June |  |  |
| - 7 | - July |  |  |
| - 8 | - August |  |  |
| - 9 | - September |  |  |
| - 10 | - October |  |  |
| - 11 | - November |  |  |
| - 12 | - December |  |  |
| - 13 |  |  |  |
| - 14 |  |  |  |
| - 15 |  |  |  |
| - 16 |  |  |  |
| - 17 |  |  |  |
| - 18 |  |  |  |
| - 19 |  |  |  |
| - 20 |  |  |  |
| - 21 |  |  |  |
| - 22 |  |  |  |
| - 23 |  |  |  |
| - 24 |  |  |  |
| - 25 |  |  |  |
| - 26 |  |  |  |
| - 27 |  |  |  |
| - 28 |  |  |  |
| - 29 |  |  |  |
| - 30 |  |  |  |
| - 31 |  |  |  |

**Did you experience any of the following symptoms in the period after the first vaccination?**

|  | No | Yes, mild symptoms | Yes, moderate symptoms | Yes,  severe symptoms |
| --- | --- | --- | --- | --- |
| Redness and/or pain at the injection site |  |  |  |  |
| Skin rash |  |  |  |  |
| Nausea |  |  |  |  |
| Vomiting |  |  |  |  |
| Fever |  |  |  |  |
| Shivering/chills |  |  |  |  |
| Tiredness |  |  |  |  |
| General malaise |  |  |  |  |
| Joint pain |  |  |  |  |
| Muscle pain |  |  |  |  |
| Headache |  |  |  |  |
| Diarrhoea |  |  |  |  |
| Dizziness |  |  |  |  |
| Urge to sleep/fatigue |  |  |  |  |
| Swollen lymph nodes |  |  |  |  |
| Facial swelling |  |  |  |  |
| Facial paralysis |  |  |  |  |
| Pain in the arms and legs |  |  |  |  |
| Allergic reaction |  |  |  |  |
| Shortness of breath |  |  |  |  |
| Bruising/bleeding under the skin |  |  |  |  |

**If you experienced symptoms that are not listed above, you can enter them here:**

*Please notice that the comments are not read on a continuous basis. Thus, if you have any questions or expect a response, we refer to our email bicovac@ph.au.dk*.

___________________________________________

**When did you receive the second vaccine?**

*If you do not remember the exact day you received the 2nd vaccine, please just state the month you were vaccinated.*

| Day | Month | Year |  |
| --- | --- | --- | --- |
| - 1 | - January | - 2021 | - Do not remember |
| - 2 | - February | - 2020 |  |
| - 3 | - March | - 2019 |  |
| - 4 | - April |  |  |
| - 5 | - May |  |  |
| - 6 | - June |  |  |
| - 7 | - July |  |  |
| - 8 | - August |  |  |
| - 9 | - September |  |  |
| - 10 | - October |  |  |
| - 11 | - November |  |  |
| - 12 | - December |  |  |
| - 13 |  |  |  |
| - 14 |  |  |  |
| - 15 |  |  |  |
| - 16 |  |  |  |
| - 17 |  |  |  |
| - 18 |  |  |  |
| - 19 |  |  |  |
| - 20 |  |  |  |
| - 21 |  |  |  |
| - 22 |  |  |  |
| - 23 |  |  |  |
| - 24 |  |  |  |
| - 25 |  |  |  |
| - 26 |  |  |  |
| - 27 |  |  |  |
| - 28 |  |  |  |
| - 29 |  |  |  |
| - 30 |  |  |  |
| - 31 |  |  |  |

**Did you experience any of the following symptoms in the period after the second vaccination?**

|  | No | Yes, mild symptoms | Yes, moderate symptoms | Yes,  severe symptoms |
| --- | --- | --- | --- | --- |
| Redness and/or pain at the injection site |  |  |  |  |
| Skin rash |  |  |  |  |
| Nausea |  |  |  |  |
| Vomiting |  |  |  |  |
| Fever |  |  |  |  |
| Shivering/chills |  |  |  |  |
| Tiredness |  |  |  |  |
| General malaise |  |  |  |  |
| Joint pain |  |  |  |  |
| Muscle pain |  |  |  |  |
| Headache |  |  |  |  |
| Diarrhoea |  |  |  |  |
| Dizziness |  |  |  |  |
| Urge to sleep/fatigue |  |  |  |  |
| Swollen lymph nodes |  |  |  |  |
| Facial swelling |  |  |  |  |
| Facial paralysis |  |  |  |  |
| Pain in the arms and legs |  |  |  |  |
| Allergic reaction |  |  |  |  |
| Shortness of breath |  |  |  |  |
| Bruising/bleeding under the skin |  |  |  |  |

**If you experienced symptoms that are not listed above, you can enter them here:**

*Please notice that the comments are not read on a continuous basis. Thus, if you have any questions or expect a response, we refer to our email bicovac@ph.au.dk*.

___________________________________________

**Questions about infections**

*The following questions concern infections.*

**Have you since you answered the first questionnaire been infected with Corona?**

- No, I do not think I have been infected with COVID-19
- Yes, I think/know that I have been infected with COVID-19

**When did you test positive?**

*Please submit date.* *If you do not remember the exact day you tested positive for COVID-19, please just state the month and year you tested positive.*

| Day | Month | Year |  |
| --- | --- | --- | --- |
| - 1 | - January | - 2021 | - I was not tested |
| - 2 | - February | - 2020 | - Don’t remember |
| - 3 | - March | - 2019 |  |
| - 4 | - April |  |  |
| - 5 | - May |  |  |
| - 6 | - June |  |  |
| - 7 | - July |  |  |
| - 8 | - August |  |  |
| - 9 | - September |  |  |
| - 10 | - October |  |  |
| - 11 | - November |  |  |
| - 12 | - December |  |  |
| - 13 |  |  |  |
| - 14 |  |  |  |
| - 15 |  |  |  |
| - 16 |  |  |  |
| - 17 |  |  |  |
| - 18 |  |  |  |
| - 19 |  |  |  |
| - 20 |  |  |  |
| - 21 |  |  |  |
| - 22 |  |  |  |
| - 23 |  |  |  |
| - 24 |  |  |  |
| - 25 |  |  |  |
| - 26 |  |  |  |
| - 27 |  |  |  |
| - 28 |  |  |  |
| - 29 |  |  |  |
| - 30 |  |  |  |
| - 31 |  |  |  |

**Did you experience symptoms of the COVID-19 infection?**

- No
- Yes, but only mild symptoms
- Yes, moderate symptoms
- Yes, severe symptoms but without hospitalisation
- Yes, severe symptoms that resulted in hospitalisation

**Have you since you answered the first questionnaire had other infections aside from COVID-19?**

*For example, a cold, tonsillitis or bladder infections.*

- No
- Yes

**Questions about health, well-being, leisure time and lifestyle**

**In general, would you say your health is:**

| Excellent | Very good | Good | Fair | Poor |
| --- | --- | --- | --- | --- |
|  |  |  |  |  |

**Does your health now limit you in moderate activities such as moving a table, vacuuming or riding a bike?**

| No, not limited at all | Yes, slightly limited | Yes, very limited |
| --- | --- | --- |
|  |  |  |

**Does your health now limit you in climbing several flights of stairs?**

| No, not limited  at all | Yes, slightly limited | Yes, very limited |
| --- | --- | --- |
|  |  |  |

**During the past four weeks, have you had any of the following problems with your work or other regular daily activities as a result of your physical health?**

|  | At no point in time | Occasionally | Some of the time | Most  of the time | All the time |
| --- | --- | --- | --- | --- | --- |
| I accomplished less than I would like |  |  |  |  |  |
| I have been limited in the kind of work or other activities I have been able to perform |  |  |  |  |  |

**During the past four weeks, have you had any of the following problems with your work or other regular daily activities as a result of any emotional problems?**

|  | At no point in time | Occasionally | Some of the time | Most  of the time | All the time |
| --- | --- | --- | --- | --- | --- |
| I accomplished less than I would like |  |  |  |  |  |
| I didn´t do work or other activities as carefully as usual |  |  |  |  |  |

**During the past 4 weeks, how much did physical pain interfere with your normal work (both work outside the home and housework)?**

| Not at all | A little bit | Moderately | Quite a bit | Extremely |
| --- | --- | --- | --- | --- |
|  |  |  |  |  |

**Questions about symptoms you have been bothered by over the past four weeks**

**During the past four weeks, how much have you been bothered by?**

Symptoms from the heart and lungs

|  | Not at all | A bit | Somewhat | Quite  a bit | A lot |
| --- | --- | --- | --- | --- | --- |
| Palpitations or heart pounding? |  |  |  |  |  |
| Precordial discomfort? |  |  |  |  |  |
| Breathlessness without exertion? |  |  |  |  |  |
| Hyperventilation? |  |  |  |  |  |
| Hot or cold sweats? |  |  |  |  |  |
| Dry mouth? |  |  |  |  |  |

Symptoms from the stomach and the intestines

|  | Not at all | A bit | Somewhat | Quite  a bit | A lot |
| --- | --- | --- | --- | --- | --- |
| Frequent, loose bowel movements? |  |  |  |  |  |
| Abdominal pain? |  |  |  |  |  |
| Feeling bloated/full of gas/distended? |  |  |  |  |  |
| Diarrhoea? |  |  |  |  |  |
| Regurgitations? |  |  |  |  |  |
| Nausea? |  |  |  |  |  |
| Burning sensation of the chest or upper part of the stomach/epigastrium? |  |  |  |  |  |

Symptoms from muscles and joints

|  | Not at all | A bit | Somewhat | Quite  a bit | A lot |
| --- | --- | --- | --- | --- | --- |
| Pain in arms or legs? |  |  |  |  |  |
| Muscular aches or pain? |  |  |  |  |  |
| Pain in the joints? |  |  |  |  |  |
| Feeling of paralysis in the arms or legs? |  |  |  |  |  |
| Back ache? |  |  |  |  |  |
| Pain moving from one place to another? |  |  |  |  |  |
| Unpleasant numbness or tingling sensations? |  |  |  |  |  |

General symptoms

|  | Not at all | A bit | Somewhat | Quite  a bit | A lot |
| --- | --- | --- | --- | --- | --- |
| Concentration difficulties? |  |  |  |  |  |
| Excessive fatigue? |  |  |  |  |  |
| Headache? |  |  |  |  |  |
| Impairment of memory? |  |  |  |  |  |
| Dizziness? |  |  |  |  |  |

Other symptoms

|  | Not at all | A bit | Somewhat | Quite  a bit | A lot |
| --- | --- | --- | --- | --- | --- |
| Involuntary muscle movements/convulsions? |  |  |  |  |  |
| Sleep disturbances? |  |  |  |  |  |
| Visual disturbances? |  |  |  |  |  |
| Tinnitus? |  |  |  |  |  |
| Loss of sense of smell? |  |  |  |  |  |
| Loss of sense of taste? |  |  |  |  |  |

**In the most recent questions, you have ticked one or more physical symptoms in the last four weeks.**

**How much does your symptoms affect your life?**

| No affect at all | |  |  |  |  |  |  |  |  | Severely affects my life | |
| --- | --- | --- | --- | --- | --- | --- | --- | --- | --- | --- | --- |
| - 1 | - 2 | | - 3 | - 4 | - 5 | - 6 | - 7 | - 8 | - 9 | | - 10 |

**How long do you think your symptoms will continue?**

| A very short time | |  |  |  |  |  |  |  |  | Forever | |
| --- | --- | --- | --- | --- | --- | --- | --- | --- | --- | --- | --- |
| - 1 | - 2 | | - 3 | - 4 | - 5 | - 6 | - 7 | - 8 | - 9 | | - 10 |

**How much control do you feel you have over your symptoms?**

| Absolutely no control | |  |  |  |  |  |  |  |  | Extreme amount of control | |
| --- | --- | --- | --- | --- | --- | --- | --- | --- | --- | --- | --- |
| - 1 | - 2 | | - 3 | - 4 | - 5 | - 6 | - 7 | - 8 | - 9 | | - 10 |

**How much do you think that treatment can help your symptoms?**

| Not at all | |  |  |  |  |  |  |  |  | Extremely helpful | |
| --- | --- | --- | --- | --- | --- | --- | --- | --- | --- | --- | --- |
| - 1 | - 2 | | - 3 | - 4 | - 5 | - 6 | - 7 | - 8 | - 9 | | - 10 |

**How concerned are you about your symptoms?**

| Not at all concerned | |  |  |  |  |  |  |  |  | Extremely concerned | |
| --- | --- | --- | --- | --- | --- | --- | --- | --- | --- | --- | --- |
| - 1 | - 2 | | - 3 | - 4 | - 5 | - 6 | - 7 | - 8 | - 9 | | - 10 |

**How well do you feel you understand your symptoms?**

| Do not understand them at all | |  |  |  |  |  |  |  |  | Understand them very clearly | |
| --- | --- | --- | --- | --- | --- | --- | --- | --- | --- | --- | --- |
| - 1 | - 2 | | - 3 | - 4 | - 5 | - 6 | - 7 | - 8 | - 9 | | - 10 |

**How much does your symptoms affect you emotionally? (I.e. do they make you feel angry, afraid, restless or depressed?)**

| Not at all affected emotionally | |  |  |  |  |  |  |  |  | Extremely affected emotionally | |
| --- | --- | --- | --- | --- | --- | --- | --- | --- | --- | --- | --- |
| - 1 | - 2 | | - 3 | - 4 | - 5 | - 6 | - 7 | - 8 | - 9 | | - 10 |

**Questions of fatigue and exhaustion**

**Throughout the past four weeks, how much have you been bothered by fatigue and exhaustion?**

|  | Symptom not present | Mild | Moderate | Severe | Very severe |
| --- | --- | --- | --- | --- | --- |
| Dead, heavy feeling after starting to exercise |  |  |  |  |  |
| Next day soreness or fatigue after non-strenuous, everyday activities |  |  |  |  |  |
| Mentally tired after the slightest effort |  |  |  |  |  |
| Physically tired after minimum exercise |  |  |  |  |  |
| Physically drained or sick after light activity |  |  |  |  |  |

**If there is information you feel you need to elaborate on, you can do so in the comments section here.**

*Please notice that the comments are not read on a continuous basis. Thus, if you have any questions or expect a response, we refer to our email* [*bicovac@ph.au.dk*](mailto:bicovac@ph.au.dk)*.*

___________________________________________

**That was the final question.**

Thank you for taking the time to answer the questionnaire.

When you close the questionnaire, it will no longer be possible to change your answers.

We will send you a short questionnaire in a few weeks.
