# Supplementary material for: Cohort profile: The BiCoVac cohort - a nationwide Danish cohort to assess short and long-term symptoms following COVID-19 vaccination
Source: Eur J Epidemiol. 2025 Feb 7;40(2):225–33. doi: 10.1007/s10654-025-01204-1 (PMC12018486; doi:10.1007/s10654-025-01204-1)
Supplement: Supplementary file 4 — Supplementary Material 4 [file 10654_2025_1204_MOESM4_ESM.docx]

**Supplementary File S4 – Third follow-up questionnaire**

**Title:** Cohort Profile: The BiCoVac Cohort - a nationwide Danish cohort to assess short and long-term symptoms following COVID-19 vaccination

**Journal name:** European Journal of Epidemiology

**Authors:** Christina Bisgaard Jensen, Kristoffer Torp Hansen, Casper Mailund Nielsen, Stefan Nygaard Hansen, Henrik Nielsen, Charlotte Ulrika Rask, Per Fink, Thomas Meinertz Dantoft, Torben Jørgensen, Bodil Hammer Bech, Sanne Møller Thysen, Dorte Rytter

**Affiliation of the corresponding author:** Department of Public Health, Aarhus University, DK-8000 Aarhus, Denmark

**E-mail of the corresponding author:** cbj@ph.au.dk

**Dear participant**

Thank you for accepting to complete one more questionnaire in our study about corona vaccinations. All your answers provide important knowledge which can contribute to understanding whether corona vaccines are associated with side effects.

Your answers are important - whether you are healthy or sick - and whether you have been / are planning to be vaccinated or not. We ask you to complete all questions as well as you can.

When you begin answering the questions in the electronic questionnaire, your answers will regularly be saved and will thus be included in the study regardless of whether the questionnaire is fully or partially completed. However, you can change your answers prior to clicking on "Finish".

**Questions about vaccination against COVID-19**

*The following questions concern vaccination against COVID-19.*

**How many vaccines against COVID-19 have you received?**

- 0
- 1
- 2
- 3
- 4

**When did you receive the first vaccine?**

*If you do not remember the exact day, you received the 1^st^ vaccine, please just state the month you were vaccinated.*

| Day | Month | Year |  |
| --- | --- | --- | --- |
| - 1 | - January | - 2021 | - Do not remember |
| - 2 | - February | - 2020 |  |
| - 3 | - March | - 2019 |  |
| - 4 | - April |  |  |
| - 5 | - May |  |  |
| - 6 | - June |  |  |
| - 7 | - July |  |  |
| - 8 | - August |  |  |
| - 9 | - September |  |  |
| - 10 | - October |  |  |
| - 11 | - November |  |  |
| - 12 | - December |  |  |
| - 13 |  |  |  |
| - 14 |  |  |  |
| - 15 |  |  |  |
| - 16 |  |  |  |
| - 17 |  |  |  |
| - 18 |  |  |  |
| - 19 |  |  |  |
| - 20 |  |  |  |
| - 21 |  |  |  |
| - 22 |  |  |  |
| - 23 |  |  |  |
| - 24 |  |  |  |
| - 25 |  |  |  |
| - 26 |  |  |  |
| - 27 |  |  |  |
| - 28 |  |  |  |
| - 29 |  |  |  |
| - 30 |  |  |  |
| - 31 |  |  |  |

**Which vaccine did you receive as your first dose?**

- Pfizer-BioNTech
- Moderna
- AstraZeneca
- Johnson & Johnson
- Other: _____________________________________
- Don’t know

**Did you experience any of the following symptoms in the period after the first vaccination?**

|  | No | Yes, mild symptoms | Yes, moderate symptoms | Yes,  severe symptoms |
| --- | --- | --- | --- | --- |
| Redness and/or pain at the injection site |  |  |  |  |
| Skin rash |  |  |  |  |
| Nausea |  |  |  |  |
| Vomiting |  |  |  |  |
| Fever |  |  |  |  |
| Shivering/chills |  |  |  |  |
| Tiredness |  |  |  |  |
| General malaise |  |  |  |  |
| Joint pain |  |  |  |  |
| Muscle pain |  |  |  |  |
| Headache |  |  |  |  |
| Diarrhoea |  |  |  |  |
| Dizziness |  |  |  |  |
| Urge to sleep/fatigue |  |  |  |  |
| Swollen lymph nodes |  |  |  |  |
| Facial swelling |  |  |  |  |
| Facial paralysis |  |  |  |  |
| Pain in the arms and legs |  |  |  |  |
| Allergic reaction |  |  |  |  |
| Shortness of breath |  |  |  |  |
| Bruising/bleeding under the skin |  |  |  |  |

**If you experienced symptoms that are not listed above, you can enter them here:**

*Please notice that the comments are not read on a continuous basis. Thus, if you have any questions or expect a response, we refer to our email bicovac@ph.au.dk*.

___________________________________________

**When did you receive the second vaccine?**

*If you do not remember the exact day you received the 2^nd^ vaccine, please just state the month you were vaccinated.*

| Day | Month | Year |  |
| --- | --- | --- | --- |
| - 1 | - January | - 2021 | - Do not remember |
| - 2 | - February | - 2020 |  |
| - 3 | - March | - 2019 |  |
| - 4 | - April |  |  |
| - 5 | - May |  |  |
| - 6 | - June |  |  |
| - 7 | - July |  |  |
| - 8 | - August |  |  |
| - 9 | - September |  |  |
| - 10 | - October |  |  |
| - 11 | - November |  |  |
| - 12 | - December |  |  |
| - 13 |  |  |  |
| - 14 |  |  |  |
| - 15 |  |  |  |
| - 16 |  |  |  |
| - 17 |  |  |  |
| - 18 |  |  |  |
| - 19 |  |  |  |
| - 20 |  |  |  |
| - 21 |  |  |  |
| - 22 |  |  |  |
| - 23 |  |  |  |
| - 24 |  |  |  |
| - 25 |  |  |  |
| - 26 |  |  |  |
| - 27 |  |  |  |
| - 28 |  |  |  |
| - 29 |  |  |  |
| - 30 |  |  |  |
| - 31 |  |  |  |

**Which vaccine did you receive as your second dose?**

- Pfizer-BioNTech
- Moderna
- AstraZeneca
- Johnson & Johnson
- Other: _____________________________________
- Don’t know

**Did you experience any of the following symptoms in the period after the second vaccination?**

|  | No | Yes, mild symptoms | Yes, moderate symptoms | Yes,  severe symptoms |
| --- | --- | --- | --- | --- |
| Redness and/or pain at the injection site |  |  |  |  |
| Skin rash |  |  |  |  |
| Nausea |  |  |  |  |
| Vomiting |  |  |  |  |
| Fever |  |  |  |  |
| Shivering/chills |  |  |  |  |
| Tiredness |  |  |  |  |
| General malaise |  |  |  |  |
| Joint pain |  |  |  |  |
| Muscle pain |  |  |  |  |
| Headache |  |  |  |  |
| Diarrhoea |  |  |  |  |
| Dizziness |  |  |  |  |
| Urge to sleep/fatigue |  |  |  |  |
| Swollen lymph nodes |  |  |  |  |
| Facial swelling |  |  |  |  |
| Facial paralysis |  |  |  |  |
| Pain in the arms and legs |  |  |  |  |
| Allergic reaction |  |  |  |  |
| Shortness of breath |  |  |  |  |
| Bruising/bleeding under the skin |  |  |  |  |

**If you experienced symptoms that are not listed above, you can enter them here:**

*Please notice that the comments are not read on a continuous basis. Thus, if you have any questions or expect a response, we refer to our email bicovac@ph.au.dk*.

___________________________________________

**When did you receive the third vaccine?**

*If you do not remember the exact day you received the* 3^nd^ *vaccine, please just state the month you were vaccinated.*

| Day | Month | Year |  |
| --- | --- | --- | --- |
| - 1 | - January | - 2021 | - Do not remember |
| - 2 | - February | - 2020 |  |
| - 3 | - March | - 2019 |  |
| - 4 | - April |  |  |
| - 5 | - May |  |  |
| - 6 | - June |  |  |
| - 7 | - July |  |  |
| - 8 | - August |  |  |
| - 9 | - September |  |  |
| - 10 | - October |  |  |
| - 11 | - November |  |  |
| - 12 | - December |  |  |
| - 13 |  |  |  |
| - 14 |  |  |  |
| - 15 |  |  |  |
| - 16 |  |  |  |
| - 17 |  |  |  |
| - 18 |  |  |  |
| - 19 |  |  |  |
| - 20 |  |  |  |
| - 21 |  |  |  |
| - 22 |  |  |  |
| - 23 |  |  |  |
| - 24 |  |  |  |
| - 25 |  |  |  |
| - 26 |  |  |  |
| - 27 |  |  |  |
| - 28 |  |  |  |
| - 29 |  |  |  |
| - 30 |  |  |  |
| - 31 |  |  |  |

**Which vaccine did you receive as your third dose?**

- Pfizer-BioNTech
- Moderna
- AstraZeneca
- Johnson & Johnson
- Other: _____________________________________
- Don’t know

**Did you experience any of the following symptoms in the period after the third vaccination?**

|  | No | Yes, mild symptoms | Yes, moderate symptoms | Yes,  severe symptoms |
| --- | --- | --- | --- | --- |
| Redness and/or pain at the injection site |  |  |  |  |
| Skin rash |  |  |  |  |
| Nausea |  |  |  |  |
| Vomiting |  |  |  |  |
| Fever |  |  |  |  |
| Shivering/chills |  |  |  |  |
| Tiredness |  |  |  |  |
| General malaise |  |  |  |  |
| Joint pain |  |  |  |  |
| Muscle pain |  |  |  |  |
| Headache |  |  |  |  |
| Diarrhoea |  |  |  |  |
| Dizziness |  |  |  |  |
| Urge to sleep/fatigue |  |  |  |  |
| Swollen lymph nodes |  |  |  |  |
| Facial swelling |  |  |  |  |
| Facial paralysis |  |  |  |  |
| Pain in the arms and legs |  |  |  |  |
| Allergic reaction |  |  |  |  |
| Shortness of breath |  |  |  |  |
| Bruising/bleeding under the skin |  |  |  |  |

**If you experienced symptoms that are not listed above, you can enter them here:**

*Please notice that the comments are not read on a continuous basis. Thus, if you have any questions or expect a response, we refer to our email bicovac@ph.au.dk*.

___________________________________________

**When did you receive the fourth vaccine?**

*If you do not remember the exact day you received the* 4^th^ *vaccine, please just state the month you were vaccinated.*

| Day | Month | Year |  |
| --- | --- | --- | --- |
| - 1 | - January | - 2021 | - Do not remember |
| - 2 | - February | - 2020 |  |
| - 3 | - March | - 2019 |  |
| - 4 | - April |  |  |
| - 5 | - May |  |  |
| - 6 | - June |  |  |
| - 7 | - July |  |  |
| - 8 | - August |  |  |
| - 9 | - September |  |  |
| - 10 | - October |  |  |
| - 11 | - November |  |  |
| - 12 | - December |  |  |
| - 13 |  |  |  |
| - 14 |  |  |  |
| - 15 |  |  |  |
| - 16 |  |  |  |
| - 17 |  |  |  |
| - 18 |  |  |  |
| - 19 |  |  |  |
| - 20 |  |  |  |
| - 21 |  |  |  |
| - 22 |  |  |  |
| - 23 |  |  |  |
| - 24 |  |  |  |
| - 25 |  |  |  |
| - 26 |  |  |  |
| - 27 |  |  |  |
| - 28 |  |  |  |
| - 29 |  |  |  |
| - 30 |  |  |  |
| - 31 |  |  |  |

**Which vaccine did you receive as your fourth dose?**

- Pfizer-BioNTech
- Moderna
- AstraZeneca
- Johnson & Johnson
- Other: _____________________________________
- Don’t know

**Did you experience any of the following symptoms in the period after the fourth vaccination?**

|  | No | Yes, mild symptoms | Yes, moderate symptoms | Yes,  severe symptoms |
| --- | --- | --- | --- | --- |
| Redness and/or pain at the injection site |  |  |  |  |
| Skin rash |  |  |  |  |
| Nausea |  |  |  |  |
| Vomiting |  |  |  |  |
| Fever |  |  |  |  |
| Shivering/chills |  |  |  |  |
| Tiredness |  |  |  |  |
| General malaise |  |  |  |  |
| Joint pain |  |  |  |  |
| Muscle pain |  |  |  |  |
| Headache |  |  |  |  |
| Diarrhoea |  |  |  |  |
| Dizziness |  |  |  |  |
| Urge to sleep/fatigue |  |  |  |  |
| Swollen lymph nodes |  |  |  |  |
| Facial swelling |  |  |  |  |
| Facial paralysis |  |  |  |  |
| Pain in the arms and legs |  |  |  |  |
| Allergic reaction |  |  |  |  |
| Shortness of breath |  |  |  |  |
| Bruising/bleeding under the skin |  |  |  |  |

**If you experienced symptoms that are not listed above, you can enter them here:**

*Please notice that the comments are not read on a continuous basis. Thus, if you have any questions or expect a response, we refer to our email bicovac@ph.au.dk*.

___________________________________________

**Questions about infections**

*The following questions concern infections.*

**Have you since December 1^st^ 2021 been infected with Corona?**

- No, I do not think I have been infected with Corona since December 1st, 2021
- Yes, I think/know that I have been infected with Corona since December 1st, 2021

**When did you test positive?**

*Please submit date. If you do not remember the exact day you tested positive for COVID-19, please just state the month and year you tested positive.*

| Day | Month | Year |  |
| --- | --- | --- | --- |
| - 1 | - January | - 2021 | - I was not tested |
| - 2 | - February | - 2020 | - Don’t remember |
| - 3 | - March | - 2019 |  |
| - 4 | - April |  |  |
| - 5 | - May |  |  |
| - 6 | - June |  |  |
| - 7 | - July |  |  |
| - 8 | - August |  |  |
| - 9 | - September |  |  |
| - 10 | - October |  |  |
| - 11 | - November |  |  |
| - 12 | - December |  |  |
| - 13 |  |  |  |
| - 14 |  |  |  |
| - 15 |  |  |  |
| - 16 |  |  |  |
| - 17 |  |  |  |
| - 18 |  |  |  |
| - 19 |  |  |  |
| - 20 |  |  |  |
| - 21 |  |  |  |
| - 22 |  |  |  |
| - 23 |  |  |  |
| - 24 |  |  |  |
| - 25 |  |  |  |
| - 26 |  |  |  |
| - 27 |  |  |  |
| - 28 |  |  |  |
| - 29 |  |  |  |
| - 30 |  |  |  |
| - 31 |  |  |  |

**Did you experience symptoms of the COVID-19 infection?**

- No
- Yes, but only mild symptoms
- Yes, moderate symptoms
- Yes, severe symptoms but without hospitalisation
- Yes, severe symptoms that resulted in hospitalisation

**Have you since December 1^st^, 2021 had other infections aside from Corona?**

*For example, a cold, tonsillitis or bladder infections.*

- No
- Yes
- Don’t know

**Questions about symptoms you have been bothered by over the past four weeks**

**During the past four weeks, how much have you been bothered by?**

Symptoms from the heart and lungs

|  | Not at all | A bit | Somewhat | Quite  a bit | A lot |
| --- | --- | --- | --- | --- | --- |
| Palpitations or heart pounding? |  |  |  |  |  |
| Precordial discomfort? |  |  |  |  |  |
| Breathlessness without exertion? |  |  |  |  |  |
| Hyperventilation? |  |  |  |  |  |
| Hot or cold sweats? |  |  |  |  |  |
| Dry mouth? |  |  |  |  |  |

Symptoms from the stomach and the intestines

|  | Not at all | A bit | Somewhat | Quite  a bit | A lot |
| --- | --- | --- | --- | --- | --- |
| Frequent, loose bowel movements? |  |  |  |  |  |
| Abdominal pain? |  |  |  |  |  |
| Feeling bloated/full of gas/distended? |  |  |  |  |  |
| Diarrhoea? |  |  |  |  |  |
| Regurgitations? |  |  |  |  |  |
| Nausea? |  |  |  |  |  |
| Burning sensation of the chest or upper part of the stomach/epigastrium? |  |  |  |  |  |

Symptoms from muscles and joints

|  | Not at all | A bit | Somewhat | Quite  a bit | A lot |
| --- | --- | --- | --- | --- | --- |
| Pain in arms or legs? |  |  |  |  |  |
| Muscular aches or pain? |  |  |  |  |  |
| Pain in the joints? |  |  |  |  |  |
| Feeling of paralysis in the arms or legs? |  |  |  |  |  |
| Back ache? |  |  |  |  |  |
| Pain moving from one place to another? |  |  |  |  |  |
| Unpleasant numbness or tingling sensations? |  |  |  |  |  |

General symptoms

|  | Not at all | A bit | Somewhat | Quite  a bit | A lot |
| --- | --- | --- | --- | --- | --- |
| Concentration difficulties? |  |  |  |  |  |
| Excessive fatigue? |  |  |  |  |  |
| Headache? |  |  |  |  |  |
| Impairment of memory? |  |  |  |  |  |
| Dizziness? |  |  |  |  |  |

Other symptoms

|  | Not at all | A bit | Somewhat | Quite  a bit | A lot |
| --- | --- | --- | --- | --- | --- |
| Involuntary muscle movements/convulsions? |  |  |  |  |  |
| Sleep disturbances? |  |  |  |  |  |
| Visual disturbances? |  |  |  |  |  |
| Tinnitus? |  |  |  |  |  |
| Loss of sense of smell? |  |  |  |  |  |
| Loss of sense of taste? |  |  |  |  |  |
| Shingles (herpes zoster)? |  |  |  |  |  |

**In the most recent questions, you have ticked one or more physical symptoms in the last four weeks.**

**How much does your symptoms affect your life?**

| No affect at all | |  |  |  |  |  |  |  |  | Severely affects my life | |
| --- | --- | --- | --- | --- | --- | --- | --- | --- | --- | --- | --- |
| - 1 | - 2 | | - 3 | - 4 | - 5 | - 6 | - 7 | - 8 | - 9 | | - 10 |

**How long do you think your symptoms will continue?**

| A very short time | |  |  |  |  |  |  |  |  | Forever | |
| --- | --- | --- | --- | --- | --- | --- | --- | --- | --- | --- | --- |
| - 1 | - 2 | | - 3 | - 4 | - 5 | - 6 | - 7 | - 8 | - 9 | | - 10 |

**How much control do you feel you have over your symptoms?**

| Absolutely no control | |  |  |  |  |  |  |  |  | Extreme amount of control | |
| --- | --- | --- | --- | --- | --- | --- | --- | --- | --- | --- | --- |
| - 1 | - 2 | | - 3 | - 4 | - 5 | - 6 | - 7 | - 8 | - 9 | | - 10 |

**How much do you think that treatment can help your symptoms?**

| Not at all | |  |  |  |  |  |  |  |  | Extremely helpful | |
| --- | --- | --- | --- | --- | --- | --- | --- | --- | --- | --- | --- |
| - 1 | - 2 | | - 3 | - 4 | - 5 | - 6 | - 7 | - 8 | - 9 | | - 10 |

**How concerned are you about your symptoms?**

| Not at all concerned | |  |  |  |  |  |  |  |  | Extremely concerned | |
| --- | --- | --- | --- | --- | --- | --- | --- | --- | --- | --- | --- |
| - 1 | - 2 | | - 3 | - 4 | - 5 | - 6 | - 7 | - 8 | - 9 | | - 10 |

**How well do you feel you understand your symptoms?**

| Do not understand them at all | |  |  |  |  |  |  |  |  | Understand them very clearly | |
| --- | --- | --- | --- | --- | --- | --- | --- | --- | --- | --- | --- |
| - 1 | - 2 | | - 3 | - 4 | - 5 | - 6 | - 7 | - 8 | - 9 | | - 10 |

**How much does your symptoms affect you emotionally? (I.e. do they make you feel angry, afraid, restless or depressed?)**

| Not at all affected emotionally | |  |  |  |  |  |  |  |  | Extremely affected emotionally | |
| --- | --- | --- | --- | --- | --- | --- | --- | --- | --- | --- | --- |
| - 1 | - 2 | | - 3 | - 4 | - 5 | - 6 | - 7 | - 8 | - 9 | | - 10 |

**Questions of fatigue and exhaustion**

**Throughout the past 4 weeks, how much have you been bothered by fatigue and exhaustion?**

|  | Symptom not present | Mild | Moderate | Severe | Very severe |
| --- | --- | --- | --- | --- | --- |
| Dead, heavy feeling after starting to exercise |  |  |  |  |  |
| Next day soreness or fatigue after non-strenuous, everyday activities |  |  |  |  |  |
| Mentally tired after the slightest effort |  |  |  |  |  |
| Physically tired after minimum exercise |  |  |  |  |  |
| Physically drained or sick after light activity |  |  |  |  |  |

**Questions about menstrual period**

**Have you had a menstrual period within the past three months?**

- Yes
- No
- Don’t know
- Prefer not to say

**Have your menstrual periods been regular?**

- Yes, relatively regular without the use of hormones (contraceptive pills, mini-pills, IUD (intrauterine device), etc.)
- Yes, relatively regular with the use of hormones (contraceptive pills, mini-pills, IUD (intrauterine device), etc.)
- No, my menstrual period has been/is irregular
- Don't know
- Prefer not to say

**What do you think the reason is for not having menstrual periods?**

- I have never had menstrual periods
- I am/was pregnant
- I have begun the menopause and no longer have/had menstrual periods
- I use contraception that causes/caused my menstrual period to stop
- Other

**Have you had spotting (bleeding between periods) within the last three months?**

- Yes
- No
- Don’t know
- Prefer not to say

**This was the final of the planned questionnaires in the BiCoVac study. We will however ask you permission to contact you again if this will become relevant.**

- Yes, I give permission for you to contact me again later regarding the BiCoVac study.
- No, I do not wish to be contacted again regarding the BiCoVac study.

**That was the final question.**

Thank you for taking the time to answer the questionnaire.

When you close the questionnaire, it will no longer be possible to change your answers.
