# Supplementary material for: Cohort profile: The BiCoVac cohort - a nationwide Danish cohort to assess short and long-term symptoms following COVID-19 vaccination
Source: Eur J Epidemiol. 2025 Feb 7;40(2):225–33. doi: 10.1007/s10654-025-01204-1 (PMC12018486; doi:10.1007/s10654-025-01204-1)

**Supplementary Fig. S1 – Flowchart stratified by the number of vaccines**

**Title:** Cohort Profile: The BiCoVac Cohort - a nationwide Danish cohort to assess short and long-term symptoms following COVID-19 vaccination

**Journal name:** European Journal of Epidemiology

**Authors:** Christina Bisgaard Jensen, Kristoffer Torp Hansen, Casper Mailund Nielsen, Stefan Nygaard Hansen, Henrik Nielsen, Charlotte Ulrika Rask, Per Fink, Thomas Meinertz Dantoft, Torben Jørgensen, Bodil Hammer Bech, Sanne Møller Thysen, Dorte Rytter

**Affiliation of the corresponding author:** Department of Public Health, Aarhus University, DK-8000 Aarhus, Denmark

**E-mail of the corresponding author:** cbj@ph.au.dk

**
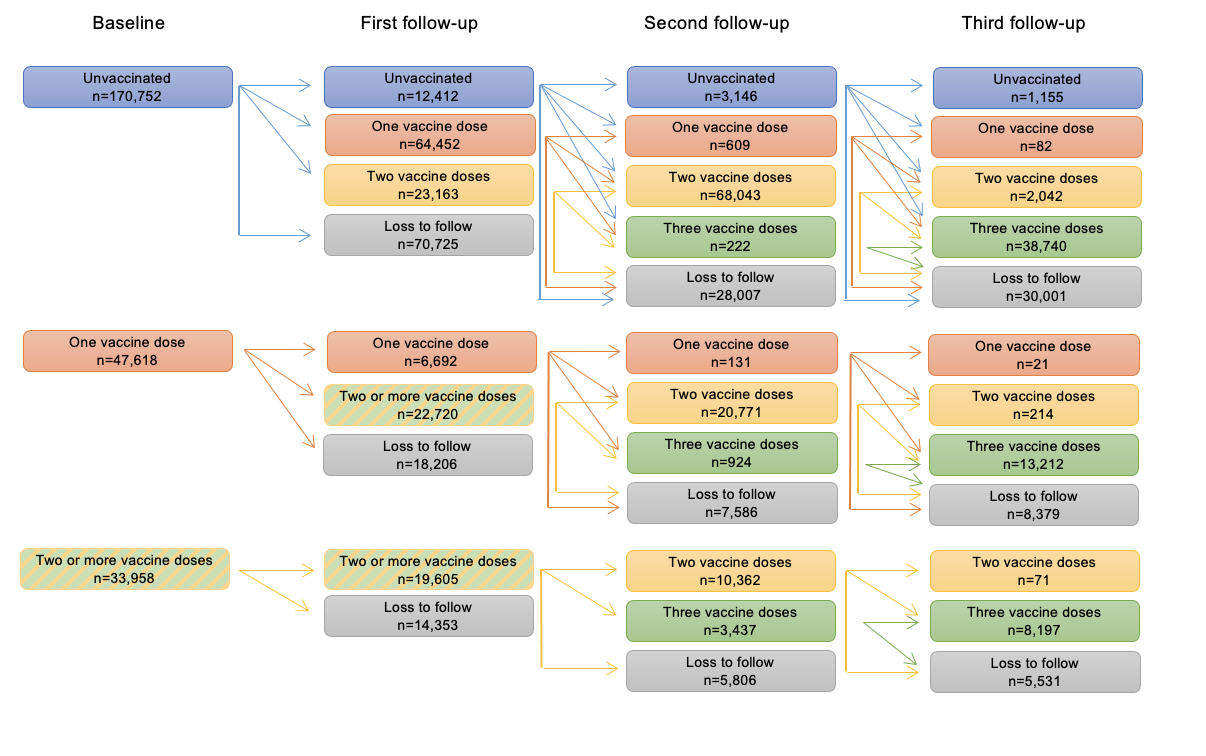
** Legend: Based on participants who initiated the questionnaires**.**


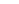

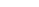

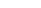

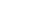

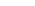

Supplement: Supplementary file 5 — Supplementary Material 5 [file 10654_2025_1204_MOESM5_ESM.docx]
